# M-MDSC *in vitro* generation from mouse bone marrow with IL-3 reveals high expression and functional activity of arginase 1

**DOI:** 10.3389/fimmu.2023.1130600

**Published:** 2023-05-19

**Authors:** Arpa Aintablian, Sandra Strozniak, Marion Heuer, Manfred B. Lutz

**Affiliations:** Institute for Virology and Immunobiology, University of Würzburg, Würzburg, Germany

**Keywords:** myeloid-derived suppressor cells (MDSC), bone marrow, IL-3, GM-CSF, *in vitro* culture, protocol

## Abstract

Myeloid-derived suppressor cells (MDSC) represent major regulators of immune responses, which can control T cells *via* their inducible nitric oxide synthase (iNOS)- and arginase 1 (Arg1)-mediated effector functions. While GM-CSF is well documented to promote MDSC development, little is known about this potential of IL-3, an established growth factor for mast cells. Here, we show that IL-3, similar to GM-CSF, generates monocytic MDSC (M-MDSC) from murine bone marrow (BM) cells after 3 days of *in vitro* culture. At this time point, predominantly CD11b^+^ CD49a^+^ monocytic and CD11b^+^ CD49a^-^ FcεR I^-^ neutrophilic cells were detectable, while CD11b^low/neg^ FcεR I^+^ mast cells accumulated only after extended culture periods. Both growth factors were equivalent in generating M-MDSC with respect to phenotype, cell yield and typical surface markers. However, IL-3 generated M-MDSC produced less TNF, IL-1β and IL-10 after activation with LPS + IFN-γ but showed higher Arg1 expression compared to GM-CSF generated M-MDSC. Arg1 was further induced together with iNOS after MDSC activation. Accordingly, an increased Arg1-dependent suppressor activity by the IL-3 generated M-MDSC was observed using respective iNOS and Arg1 inhibitors. Together, these data indicate that M-MDSC can be generated *in vitro* by IL-3, similar to GM-CSF, but with increased Arg1 expression and Arg1-mediated suppression capacity. This protocol now allows further *in vitro* studies on the role of IL-3 for MDSC biology.

## Introduction

MDSC represent mostly immature stages of granulocytic or monocytic cells that are functionally directed into an immune cell suppressing state rather than into immunogenic effector cells. Based on their expression level of Ly6C and Ly6G molecules, MDSC can be further subdivided into two subsets of M-MDSC and granulocytic MDSC (G-MDSC). The former resembling monocyte-like while the latter having mostly an immature neutrophil-like phenotype (1). MDSC use different suppressor mechanisms to exert their immunoregulatory effects. Among these the L-arginine-metabolizing enzymes iNOS and Arg1 appear most commonly reported for M-MDSC. While, iNOS induces NO release to inactivate target molecules by nitrosylation or to exert toxic effects, the up-regulation of the L-arginine transporter CAT-2B and Arg1 cause metabolic starvation in their microenvironment by L-arginine deprivation which affects proliferating T cells (2–5).

Several cytokines and growth factors contribute to MDSC generation (6–8). Among those, GM-CSF has been widely studied, since historically it gave the first clear hint as an inducer of a suppressive myeloid cell population (9). Based on these findings, we developed a protocol to generate MDSC from murine BM cells with GM-CSF *in vitro* (10). More recently, we found that GM-CSF also activates specific signaling pathways such as IRF-1 and mTOR as a ‘licensing’ step in monocytes, which is required for the acquisition of iNOS-mediated suppressor function of M-MDSC (11).

GM-CSF and IL-3 have been first described as hematopoietic growth factors that belong to the β-common family of cytokines (12). Both engage heterodimeric receptors, which consist of a specific α subunit and a shared βc subunit, ultimately forming dodecamer complexes for optimal signaling (13–15). Both cytokines exert pro-inflammatory functions (16, 17) and are therefore candidates for antibody-directed blockade of auto-inflammatory or auto-immune responses (18). On the other hand, these factors can exhibit immunosuppressive roles (19). The conditions that direct their opposite functions are not fully understood but may depend on the dose and microenvironmental factors at the anatomical site of their production (20).

IL-3 promotes the growth of pluripotent stem cells (21) and is the major growth factor that stimulates their further differentiation into mast cells *in vitro*, although it is not essential for steady state generation of mast cells *in vivo* as shown in IL-3 gene-deficient mice (22). Accordingly, murine BM cultures with IL-3 generate pure populations of mast cells after 4-6 weeks (23). We could show that IL-3 generates monocyte-derived DC (MoDC) from murine BM at culture periods of only 1 week (24), similar to our protocol using GM-CSF (25). IL-3 has been reported to skew the *in vitro* generation of macrophages towards an M2 effector phenotype by supportive signals from IL-4 or IL-13 produced by basophils (26), but also to promote immunosuppressive myeloid cells in a murine melanoma model (27). In contrast, IL-3 has been reported to be a partial inhibitor of human G-MDSC generation from CD34^+^ cells (28). Thus, there is no experimental set-up showing MDSC generation by IL-3 *in vitro* (6, 7).

Here, we describe a protocol for the generation of M-MDSC by IL-3 from murine BM cells. IL-3-generated M-MDSC show a higher Arg1 expression compared to GM-CSF-generated M-MDSC, and utilize Arg1, in addition to iNOS, for suppression of T cell proliferation. Our results reveal another method of *in vitro* generation of murine MDSC by IL-3, with enhanced Arg1 expression and suppressive capacity towards proinflammatory T cells.

## Results

### BM cultures with IL-3 or GM-CSF induce similar frequencies and progenitor proliferation *in vitro*

**Figure 1:**
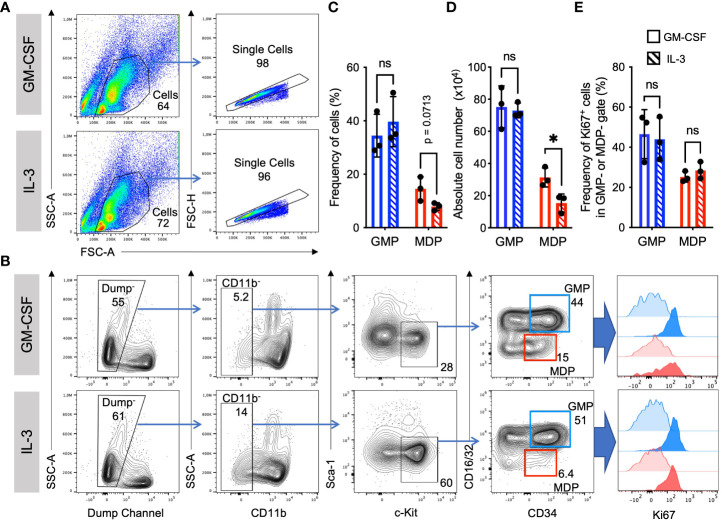
Similar frequencies and proliferation of GMP and MDP progenitors. Representative flow cytometry plots at day 3 of BM cell culture in the presence of either GM-CSF or IL-3. **(A)** Gating strategy and example stainings for GM-CSF and IL-3 cultures. **(B)** Gating strategy to detect progenitor cell proliferation with Ki67. Initial gates as shown in **(A)** Dump channel includes lineage surface markers Ter119, B220, CD4, CD8 and Ly6G. GMP were defined as CD11b^-^, Sca1^-^, CD117/c-Kit^+^, CD16/32^+^, CD34^+^; and MDP as CD11b^-^, Sca1^-^, CD117/c-Kit^+^, CD16/32^-^, CD34^+^ cells. Graphs show an assessment of the frequencies of Ki67^+^ proliferating progenitors at day 3 (strong colored histograms). Unstained cells are shown as FMO controls (weakly colored histograms). **(C)** Statistics of frequency of cells as in gates shown in **(B)**. **(D)** Absolute cell numbers per dish, calculated using frequencies as in **(C)** and live cell counts per dish in a Neubauer chamber using Trypan Blue. **(E)** Statistics of Ki67 staining as in gates shown in **(B)** Statistics by unpaired, two-tailed t-test, n=3 independent experiments. Not signi!cant (ns).

IL-3 and GM-CSF are well-known hematopoietic growth factors. To compare the role of GM-CSF and IL-3 on the expansion of myeloid cell progenitors, we analyzed their frequencies, absolute numbers, and proliferation at day 3 of BM culture with either growth factor. Elevated frequency and absolute number of granulocyte monocyte progenitors (GMP) as compared to monocyte dendritic cell progenitors (MDP) was observed in cultures with either GM-CSF or IL-3. In contrast, frequency and absolute number of MDP was slightly less in IL-3 cultures as compared to GM-CSF cultures (**Figures 1A–D**). GMP proliferated more than MDP as detected by Ki67 staining, but no significant difference could be detected between GM-CSF and IL-3 cultures (**Figures 1A, B, E**). This indicates that GM-CSF and IL-3 are comparable in promoting BM progenitor expansion *in vitro*.

### IL-3 and GM-CSF show a similar potency to generate M-MDSC phenotypes from murine BM *in vitro*

**Figure 2:**
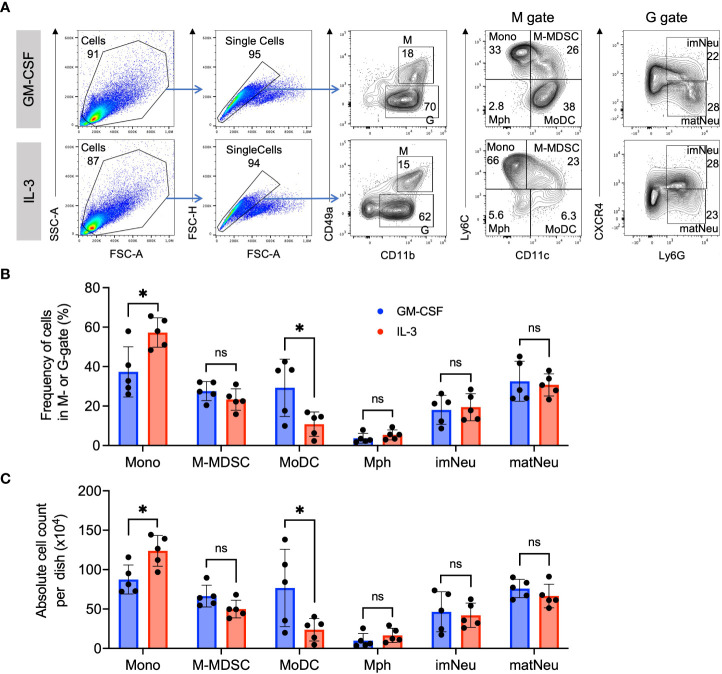
Similar phenotype and frequency of M-MDSC. **(A)** Flow cytometry example and gating strategy of day 4 BM cell cultures in the presence of either GM-CSF or IL-3. Monocytic cells (M) were defined as CD11b^+^ and CD49a^+^ and were further subdivided into Ly6C^high^ CD11c- classical monocytes (Mono), Ly6C^high^ CD11c^+^ monocytic myeloid-derived suppressor cells (M-MDSC), Ly6C^-^ CD11c^+^ dendritic cells (DC) and Ly6C^-^ CD11c^-^ macrophages (Mph). Granulocytic cells (G) were defined as CD11b^+^ and CD49a^-^ and were further subdivided into CXCR4^+^ Ly6G^+^ immature neutrophils (imNeu) and CXCR4^-^ Ly6G^+^ mature neutrophils (matNeu). Staining and gating strategy adapted from (29). **(B)** Frequencies of monocytic and granulocytic cell subsets. Gates as shown in **(A)**. **(C)** Absolute cell counts per dish, calculated using frequencies as in **(B)** and live cell counts per dish in a Neubauer chamber using Trypan Blue. Statistics compare GM-CSF with IL-3 cultures, by unpaired, two-tailed t-test, n=5 independent experiments. Not significant (ns); *p< 0.05.

We have previously established a protocol to generate preferentially M-MDSC from murine BM cells by culturing them in the presence of GM-CSF for 3 days (10, 29). Here, we applied the same procedure but used IL-3 and compared it with GM-CSF. The principal developing cells responding to these growth factors can be divided into CD11b^+^ CD49a^+^ monocytic cells (M gate) and CD11b^+^ CD49a^-^ granulocytic cells (G gate) (**Figure 2A**). Further subdivision within these gates allows the distinction of CD11b^+^ CD49a^+^ Ly6C^high^ CD11c^-^ monocytes, CD11b^+^ CD49a^+^ Ly6C^high^ CD11c^+^ M-MDSC, CD11b^+^ CD49a^+^ Ly6C^-^ CD11c^-^ macrophages, CD11b^+^ CD49a^+^ Ly6C^-^ CD11c^+^ MoDC, and CD11b^+^ CD49a^-^ CXCR4^+^ Ly6G^+^ immature and CD11b^+^ CD49a^-^ CXCR4^-^ Ly6G^+^ mature neutrophils (**Figure 2A**). Further analysis of the frequencies and absolute cell count per dish of these myeloid cell types indicated similar patterns and proportions of most subsets, except that frequencies of monocytes were higher and MoDC lower with IL-3 as compared with GM-CSF (**Figures 2B, C**). This is consistent with our previous results showing that also MoDC developed less efficient from monocytes using IL-3 as compared to GM-CSF after 1 week of culture (24). These data further support an equivalent capability of IL-3 and GM-CSF in generating M-MDSC phenotypes *in vitro*.

### Morphology and mast cell content of IL-3 and GM-CSF cultures

**Figure 3:**
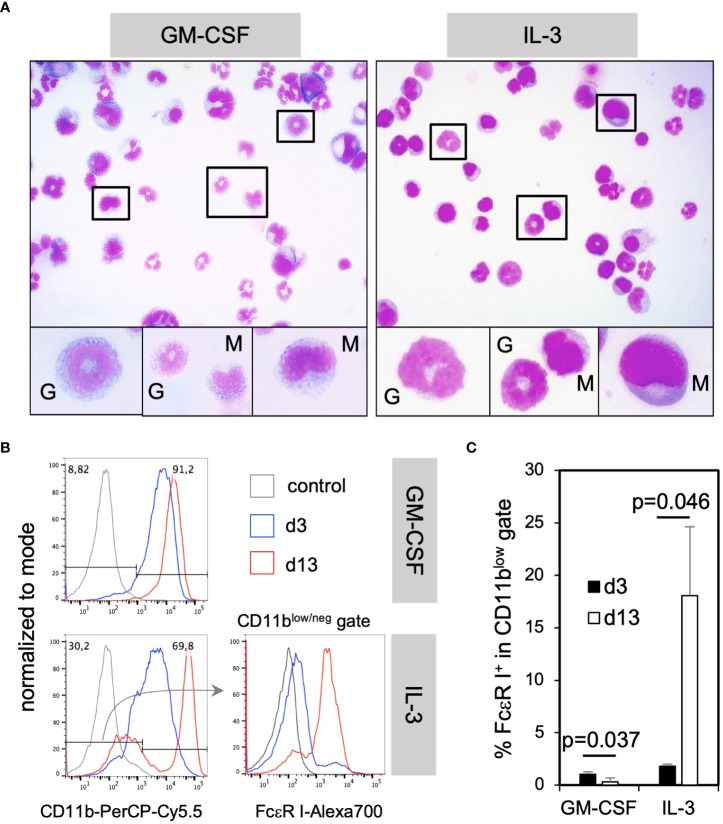
IL-3 BM cultures generate mostly neutrophilic and monocytic cells at d3 and mast cells only later during *in vitro* culture. **(A)** The morphology of monocytic and granulocytic CD11b^+^ cells cultured with GM-CSF or IL-3 for 3 days. **(B)** BM cells were cultured in GM-CSF or IL-3 for 3 or 13 days and analyzed by flow cytometry for the presence of mast cells. Gating strategy to identify CD11b^low^/^neg^ FcεR I^+^ mast cells. **(C)** Statistics of the frequency of total mast cells in GM-CSF or IL-3 cultures. Mast cells were identified by the FSC-A/SSC-A and single cell gates like in **Figure 2A** and for markers and shown in **(B)** Statistics by unpaired two-tailed t-test, n=3 independent experiments.

To further assess how GM-CSF and IL-3 cultured cells appear by morphology, cytospin preparations from these cultures were stained with H&E. Microscopic evaluation showed the presence of many cells with monocytic kidney-shaped nuclei (**Figure 3A**, “M”) or the typical ring-shaped nuclei of immature neutrophils and few mature neutrophils with segmented nuclei (**Figure 3A**, “G”) as shown before (10, 30).

IL-3 has been described as a cytokine driving mast cell generation in murine BM cultures (23). However, mast cells could not be identified by their morphology after H&E staining at day 3 (**Figure 3A**). Therefore, we tested for their presence by flow cytometry at day 3 or day 13 of culture. Mast cells were detectable as CD11b^low/neg^ FcεR I^+^ cells in cultures with IL-3 at day 13 but rarely at day 3, and were not present at any time point in GM-CSF cultures (**Figures 3B, C**). These data further corroborate that IL-3 is a potent growth factor inducing mast cell development in contrast to GM-CSF, but also that their development is slow. Remarkably, the main developing cells at early time points in day 3 cultures were neutrophilic and monocytic cells, the latter with the capacity to develop further into MoDC, as described before (24).

### Minor differences exist for expression of cytokine and chemokine receptors and signaling molecules between cultures with IL-3 and GM-CSF

Further comparative analysis was performed to assess the baseline and activation-dependent expression of cytokine and chemokine receptors on granulocytic and monocytic cell subsets promoted by either of the growth factors. Expression of GM-CSFRα (CD116) in GM-CSF cultures and that of IL-3Rα (CD123) in IL-3 cultures showed a lower trend with or without stimulation as compared to their criss-cross conditions (**Supplementary Figures 1A, B**), however they did not reach statistical significance. Also, no substantial differences were observed for the expression levels of IL-4Rα (CD124), M-CSFR (CD115) or CCR2 between IL-3 and GM-CSF cultures (**Supplementary Figures 1C–E**). Recently, we found certain signaling pathways relevant for the monocyte ‘licensing’ induced by GM-CSF which is required for MDSC development (11). To investigate whether IL-3 also activates the same molecules, we compared the baseline and activation-dependent expression levels of phosphorylated mTOR and S6 kinase. We observed no differences in the baseline as well as LPS/IFN-γ- or Zymosan-induced expression levels of both molecules between IL-3 and GM-CSF cultures (**Supplementary Figures 2A, B**). These findings suggest that there is a high similarity in the effect of IL-3 and GM-CSF on the expression of cytokine and chemokine receptors as well as signaling pathways in BM cells.

### IL-3 cultures show lower release of pro- and anti-inflammatory cytokines

**Figure 4:**
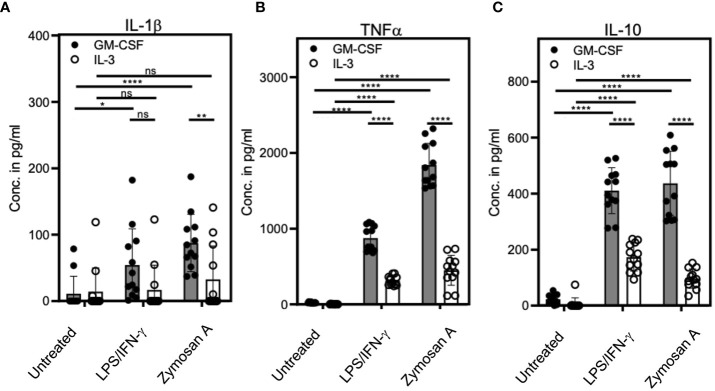
IL-3 cultures show lower cytokine release as compared with GM-CSF cultures. BM cells cultured with GM-CSF or IL-3 and were stimulated at day 3 with 0.1µg/ml LPS + 0.01ng/ml IFN-γ or 5µg/ml Zymosan. On day 4, culture supernatants were collected and ELISA was performed to detect **(A)** IL-1β, **(B)** TNF and **(C)** IL-10 concentrations. Statistics by unpaired, two-tailed t-test, n=6 independent experiments in duplicates. Not significant (ns); *p<0.05; **p<0.01; ****p<0.0001.

Next, we sought to compare the cytokine production of stimulated or unstimulated GM-CSF or IL-3 cultures. Culture supernatants were analyzed by ELISA of for the cytokines IL-1β, TNF and IL-10. Cells from LPS/IFN-γ- or Zymosan-stimulated IL-3 cultures showed a marked reduction in the release of all three cytokines as compared to those with GM-CSF (**Figures 4A–C**). This indicates that bulk IL-3 cultures are less potent in producing pro- and anti-inflammatory cytokines.

### Activation of IL-3 cultures induces higher frequencies of Arg1^+^ cells as compared with GM-CSF cultures

**Figure 5:**
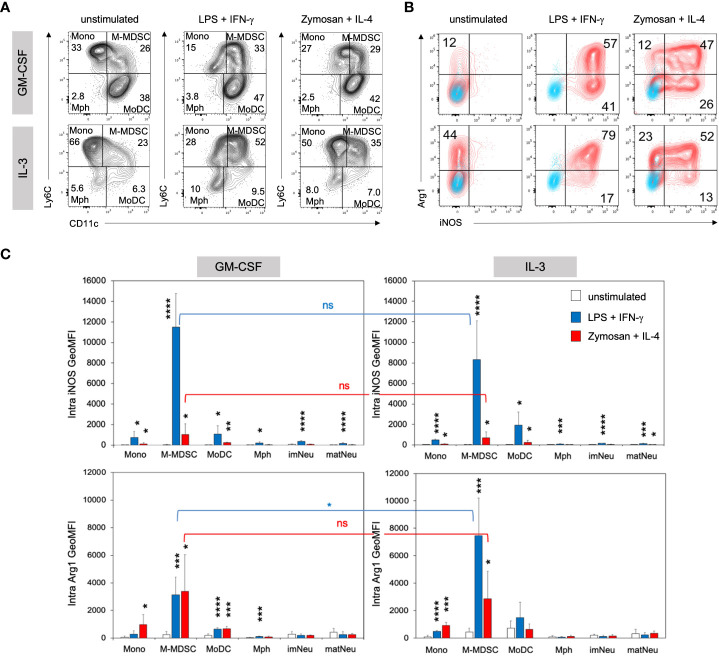
IL3-MDSC cultures induce higher Arg1 induction in M-MDSC after activation by LPS/IFN-γ. BM cells cultured in GM-CSF or IL-3 were transferred to a 24-well plate at day 3 and stimulated overnight with either 0.1µg/ml LPS + 0.01ng/ml IFN-γ or 5µg/ml Zymosan + 100U/ml IL-4 or remained unstimulated. **(A)** Flow cytometry example stainings showing quadrant gate adjustment for monocytic cell subsets (M gate) of GM-CSF or IL-3 cultured BM cells before (as in **Figure 2A**) and after stimulation at day 4. **(B)** Flow cytometry example stainings at day 4 showing the intracellular expression of iNOS and Arg1 within the M-MDSC gate. Cultures were left unstimulated or stimulated as indicated. **(C)** Statistical assessment of iNOS and Arg1 GeoMFI in monocytic and neutrophilic cell subsets of GM-CSF or IL-3 cultured BM, with or without stimulation as indicated. Gates as in **Figure 2A**. Statistics: unpaired, one-tailed t-test compared to unstimulated cells (black, only significant differences indicated); or unpaired, two-tailed t-test comparing GM-CSF and IL-3 cultures (blue, red), n=4 independent experiments. Not significant (ns); * p<0.05; ** p<0.01; *** p<0.001; **** p<0.0001.

Our previous work showed that exposure to GM-CSF for 3 days ‘licensed’ monocytes to become suppressive *via* iNOS-induced NO release. However, GM-CSF alone was not sufficient to induce iNOS expression and a second activation step by LPS/IFN-γ, among other stimulants, was required to convert these monocytes into functional M-MDSC (11). To test whether IL-3 was also able to induce licensing for generation of M-MDSC, day 3 cells cultured in the presence of either growth factor were stimulated with LPS/IFN-γ or Zymosan/IL-4 and assessed for their intracellular iNOS and Arg1 expression. Zymosan or IL-4 stimulation alone revealed lower and non-significant levels of Arg1 in IL-3 cultures and were therefore not further investigated (**Supplementary Figure 3A**). Flow cytometric analysis revealed similar phenotypes and proportions of monocytic cell subsets after LPS/IFN-γ and Zymosan/IL-4 stimulation as compared with unstimulated cultures, but quadrant gating required some adjustment (**Figure 5A**). Consistent with previous findings, iNOS expression was minimal in unstimulated IL-3 or GM-CSF cultures at day 4. However, IL-3 cultures contained higher frequencies of Arg1^+^ cells within the M-MDSC gate, which further increased after stimulation and stayed above that of GM-CSF-generated M-MDSC. The frequency of iNOS expressing cells was similar after both types of stimulation (**Figure 5B**; **Supplementary Figure 3B**). After stimulation, again the Ly6C^high^ CD11c^+^ double positive population showed the strongest iNOS and Arg1 expression per cell, identified by their GeoMFI values (**Figure 5C**). Only low levels of Arg1 expression were observed by monocytes and MoDC from IL-3 cultures (**Supplementary Figure 3B**) and were basically absent in macrophages and neutrophilic cells (**Figure 5C**; **Supplementary Figure 3B**). Thus, as found already earlier for GM-CSF cultures (29), also IL-3 cultures contain CD11b^+^ CD49a^+^ Ly6C^high^ CD11c^+^ cells representing iNOS^+^ Arg1^+^ M-MDSC (**Figure 5C**).

### IL-3 cultured M-MDSC suppress CD4^+^ and CD8^+^ T cell proliferation by increased Arg1 activity as compared with GM-CSF cultures

**Figure 6:**
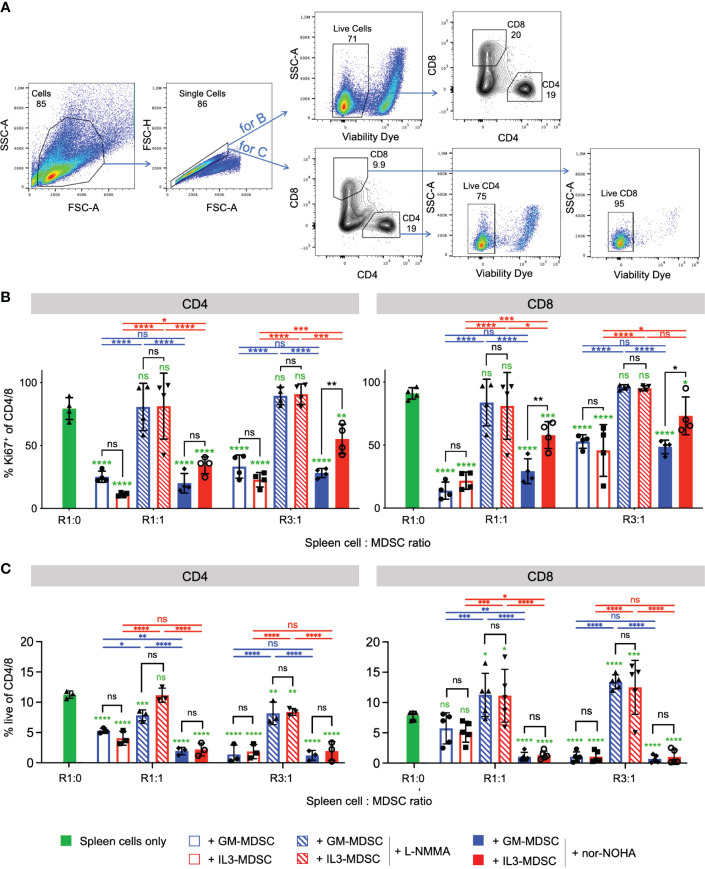
IL-3-generated MDSC show increased Arginase 1-mediated T cell suppressive capacity as compared with GM-CSF-generated MDSC Bulk spleen cells were stimulated with anti-CD3/CD28 antibodies and cultured for 3 days in the presence of GM-CSF- or IL-3-generated MDSC at the indicated ratios, and with or without iNOS inhibitor L-NMMA or Arg-1 inhibitor nor-NOHA. **(A)** Representative flow cytometry plots of bulk spleen cells at day 3 of culture with stimulating antibodies. Gating strategy to identify CD4^+^ and CD8^+^ T cells. Initial gating included FSC-A/SSC-A and doublet exclusion. For B., live cells were gated as negative for fixable viability dye incorporation. CD4^+^ and CD8^+^ lymphocytes were segregated based on their surface expression of CD4 or CD8, respectively. For C., CD4^+^ and CD8^+^ lymphocytes were segregated based on their surface expression of CD4 or CD8, respectively, and live cells within each subset were gated as negative for fixable viability dye incorporation **(B)** T cell proliferation is measured by flow cytometry and depicted as frequency of Ki67^+^ within CD4^+^ or CD8^+^ T cell subsets. **(C)** Statistics of frequency of living cells within CD4^+^ or CD8^+^ T cell subsets. Statistics was performed with two-way ANOVA and Tukey’s post test as indicated by the lines (black, blue, red) between the respective bars, and in green comparing to ‘spleen cells only’ (here with Dunnett’s post test). n=3-5 independent experiments. Not significant (ns); *p<0.05; **p<0.01; ***p<0.001; ****p<0.0001.

Since IL-3 cultures showed higher amounts of Arg1^+^ M-MDSC, we hypothesized that IL-3 generated M-MDSC should also favor Arg1-mediated suppression of activated T cells, rather than or in addition to iNOS. Therefore, we performed a suppressor assay of bulk splenocytes and assessed CD4^+^ and CD8^+^ T cell proliferation after 3 days of co-culture. Our data indicate that CD4^+^ and CD8^+^ T cell proliferation was suppressed by IL-3- or GM-CSF-generated M-MDSC at 1:1 and 3:1 spleen cell (containing the responder T cells) to M-MDSC ratios (**Figures 6A, B**). Such suppression was almost completely reverted under all conditions where the iNOS inhibitor L-NMMA was used. In addition, reversion of CD4^+^ and CD8^+^ T cell suppression by Arg1 inhibition with nor-NOHA was observed only with IL-3- but not with GM-CSF-generated M-MDSC at both ratios (**Figure 6B**). Further analysis indicates that frequencies of living CD4^+^ and CD8^+^ cells were decreased with the addition of IL-3- or GM-CSF-generated M-MDSC, and living cell frequencies were restored only when the iNOS inhibitor L-NMMA was used (**Figures 6A, C**). Next, we hypothesized whether this T cell suppression is marked by the exhaustion of T cells. Therefore, we analyzed the expression of some T cell exhaustion markers on CD4^+^ and CD8^+^ T cells after 3 days of co-culture with either GM- or IL-3-MDSC. Our results indicate no upregulation of suppression markers TIM-3, PD-1, LAG-3 and TIGIT on CD4 and CD8 T cells after co-culture with M-MDSC at both ratios (**Supplementary Figure 4**). Together, these data indicate that IL-3-generated Arg1^+^ M-MDSC utilize Arg1 to a larger extent than GM-CSF-generated M-MDSC. However, the major suppression mechanism for both growth factors remained iNOS-induced NO secretion since its inhibitor fully restored T cell proliferation under all ratios and for both CD4^+^ and CD8^+^ T cells. The suppressed T cells do not appear to be exhausted but rather undergo cell death. Thus, IL-3-generated M-MDSC express higher baseline levels of Arg1, which is further up-regulated after M-MDSC activation and accordingly plays also increased roles as a suppressor mechanism for T cell proliferation.

## Discussion

Here, we describe an alternative method to generate M-MDSC from murine BM *in vitro* by using the growth factor IL-3 instead of GM-CSF. Our results indicate that GM-CSF and IL-3 generate phenotypically and functionally highly similar M-MDSC after 3 days of culture. Both factors are equivalent in 1) driving myeloid progenitor cell differentiation and proliferation, 2) generating neutrophilic and monocytic cell subsets, 3) yielding similar frequencies of M-MDSC, 4) their surface expression of cytokine and chemokine receptors, 5) their intracellular expression of signaling molecules, and 6) their up-regulation of iNOS expression in response to LPS/IFN-γ stimulation. Major characteristics of IL-3 distinguishing them from GM-CSF cultures are 1) a less efficient conversion of monocytes into MoDC, 2) a lower production of cytokines, 3) an increased frequency and GeoMFI of Arg1 in response to LPS/IFN-γ stimulation, and 4) an increased Arg1-mediated T-cell suppression capacity of IL-3 generated M-MDSC.

Both IL-3 and GM-CSF show a high similarity in their receptor formation, signaling and function as growth factors, pro- but also anti-inflammatory cytokines. Since our own work and several other publications described GM-CSF as a potent factor to promote anti-inflammatory MDSC generation *in vitro* (10, 29, 31). Other protocols suggested the addition of IL-6 to GM-CSF cultures to develop MDSC *in vitro* (31). However, in this and another paper (32) the available direct comparisons suggest rather quantitative and not qualitative differences between the MDSC generated with or without IL-6. Since the use of IL-6 alone did not reveal any increase on MDSC frequency or allow to generate suppressive cells (31), and injection of an IL-6 blocking antibody could not reduce MDSC frequencies or an increase in CD8^+^ T cells in a melanoma model (32), we conclude that GM-CSF is the primary driver of M-MDSC generation and IL-6 fulfills the function of a secondary enhancer. Therefore, we restricted our protocols here to the comparisons of GM-CSF and IL-3 only. However, the role of IL-6 for MDSC generation is not fully clear and rewards further investigations.

Here, we tested here whether IL-3 can exert similar differentiation and functions as compared with GM-CSF. There is no formal demonstration so far that M-MDSC can be generated by IL-3 from BM cells *in vitro*. Our data reveal broad phenotypical and functional overlap between BM generated M-MDSC by both growth factors. Both IL-3 and GM-CSF triggered the same progenitors to expand and mediated their differentiation into monocytic and neutrophilic cells.

In this respect, it is of note that at day 3 of culture neutrophil generation could be observed with IL-3, highly similar to GM-CSF cultures. This was somewhat unexpected since IL-3 has been historically described as an *in vitro* and *in vivo* growth factor for human and mouse basophils and mast cells (23, 33, 34). Interestingly, IL-3-deficient mice did not show altered frequencies of mast cells and basophils, pointing to another growth factor providing their differentiation signals during steady state (22). IL-3 was also not required for the steady state development of neutrophils. However, after malaria infection, lower percentages of neutrophils were induced as compared to wild-type mice (35). This points towards IL-3 as a growth factor that expands pro-inflammatory neutrophils only under inflammatory conditions. Similarly, IL-3 transgenic mice showed a high pro-inflammatory phenotype with autoimmune and motoneural degeneration symptoms (36). More recent data indicated that during sepsis in mice IL-3 expands monocytes and neutrophils, leading to a pro-inflammatory cytokine storm, features that were not observed in IL-3-gene deficient mice which were in fact protected from sepsis (37). Thus, our 3-day *in vitro* culture method may reflect the emergency myelopoiesis function of IL-3.

The responsiveness of IL-3 or GM-CSF cultures to LPS/IFN-γ stimulation was comparable and resulted in equivalent iNOS induction. Expression of iNOS and the subsequent release of NO has been described to be one of the most prominent immunosuppressive mechanisms of M-MDSC (6, 8). Our results support our previous finding that CD11b^+^ Ly6C^+^ CD11c^+^ M-MDSC are the major iNOS expressing cell subset in GM-CSF (11) but here also in IL-3 cultures. Such activation of suppressive function was only accomplished after a 3-day priming/licensing with IL-3, similar as with GM-CSF, followed by a short microbial/cytokine stimulation as described before (11).

We observed a lower conversion rate of monocytes into MoDC in IL-3 cultures. This is in harmony with our previous findings that 8-days BM cultures with IL-3 differentiate less efficient into BM-DC as compared with GM-CSF as a growth factor (24). Similarly, IL-3 could not mimic GM-CSF to produce Langerhans cells from human blood monocytes with TGF-β1 and Delta-1 (38). However, the frequencies of M-MDSC at earlier time points in day 3 cultures were equivalent. We did not investigate the reasons for this here, because of our focus on M-MDSC generation. However, it is tempting to speculate whether this could indicate that M-MDSC develop along a separate developmental pathway from monocytes as compared with MoDC. Previously, we have shown that fully differentiated classical Ly6C^high^ mouse or CD14^+^ human monocytes can be directly converted to M-MDSC when pre-exposed to GM-CSF, an imprinting we termed ‘monocyte licensing’ (11). In addition, within the pool of BM cells differential imprinting of stem and progenitor monocytic cells by GM-CSF or IL-3 may impose their development into either direction but to different extents. The lower cytokine production of the cultures measured by ELISA correlates with the lower frequencies of MoDC generated by IL-3 culture. Since the cytokines that we analyzed are produced typically by DC in high amounts, but M-MDSC showed a similar suppression capacity from IL-3 and GM-CSF cultures, we did not investigate this further.

Mast cell and basophil activation are typical features of type 2 immune responses such as infections with helminth parasites. Therefore, IL-3, produced by T cells and basophils, has also been associated with basophilia and mastocytosis in such infections (22, 39, 40). Numerous studies support IL-3-dependent basophil activation and cytokine release in murine allergy models, highlighting the contribution of IL-3 in allergic asthma. The exposure of basophils to IL-3 has been shown to greatly increase histamine and cytokine release in response to FcεR crosslinking, promoting allergic airway hypersensitivity (41). However, this pro-inflammatory role of IL-3 has also been challenged, since in asthmatic children, a direct correlation was observed between blood IL-3 levels and an improved lung function, thereby supporting the resolution of asthma (42).

It is also long known that IL-3 enhances the IL-4 production from basophils during parasitic infections and such secretion is strongly repressed in the absence of IL-3 (43–46). Reversely, IL-4 can up-regulate the IL-3Rα expression on basophils (47), indicative for a bidirectional signal exchange. Previous findings suggested that IL-3 in the presence of IL-4 also acts on human monocytes to direct their development towards MoDC, ultimately also shifting their typically induced Th1 responses towards a Th2 direction (48). Although we did not further test for the presence or the producing cell type of IL-4 in IL-3 cultures, it is a most likely scenario that the few mast cells present already in day 3 cultures secrete considerable amounts of IL-4, which is lacking in GM-CSF cultures. This activity of IL-4 could explain the Arg1 expression observed in our day-3 IL-3 BM cultures without stimulation. This is in line with previous reports that Arg1 activity in mouse macrophages could be stimulated by IL-4 and IL-13 (49). Higher Arg1 expression has been associated with type 2 diseases, as revealed from the lungs of asthmatic animals and human patients (50). This was further corroborated in a guinea pig animal model of asthma, where application of the Arg1 inhibitor nor-NOHA fully reversed the disease (51). Furthermore, increased Arg1 activity by Th2 cytokines dampened lung immunity against bacterial infections in mice, suggesting Arg1 blockade as a therapeutic intervention to lower lung infections in asthmatic patients (52).

Arg1 is another key mechanism of immune suppression by MDSC, besides iNOS, which exerts its tolerogenic activity through L-arginine uptake and catabolism, thus depriving this amino acid from the environment and leading to T cell starvation (3, 53). Previous findings in tumor patients and murine tumor models suggested that Arg1-mediated suppressor activity may be largely exerted by G-MDSC (54, 55), while iNOS-mediated suppression is a major feature of M-MDSC (56). Moreover, the largely exclusive expression of iNOS or Arg1 by M1- or M2-polarized macrophages, respectively (57), has been explained by their competition for the common substrate L-arginine (3). It has been shown that high Arg1 expression levels promote the growth of *T. gondii* in mouse macrophages by outcompeting iNOS activity, whereas Arg1 deletion reinstalls iNOS activity and results in a better survival of the mice due to increased NO-dependent microbial killing (58). Although alternative or intermediate iNOS^+^ Arg1^+^ stages of macrophages have been identified, especially when generated culture (49), this has not been clearly dissected for M-MDSC. Our results indicate that M-MDSC can simultaneously produce both mediators of suppression upon activation with LPS/IFN-γ or Zymosan/IL-4. Interestingly, LPS/IFN-γ stimulated M-MDSC up-regulate primarily iNOS and then optionally co-induce Arg1, while Zymosan/IL-4 stimulation of M-MDSC seems to generate both types of single producers before the major frequency of M-MDSC becomes double positive for iNOS and Arg1. The latter activation process is more prominent in IL-3 cultures with a higher Arg1 level before activation. Consequently, Arg1-dependent T-cell suppression was also higher by IL-3 generated M-MDSC. These data clearly indicate that in M-MDSC the iNOS and Arg1 expression is not exclusive. Moreover, the Zymosan/IL-4 stimulation may point to the presence of differentially responsive cell subsets or first-come-first-serve signaling pathways resulting in the induction of a single enzyme in a first step before the second is co-induced. However, this requires more detailed molecular analyses.

The question remains, whether IL-3 induced MDSC play a role for tumor development. In general, IL-3 has not been investigated much in the context of tumors. Elevated serum IL-3 levels have been observed in patients with colorectal cancer as compared to healthy controls (59). In melanoma bearing mice IL-3 has been reported to bias the hematopoietic development towards a dysregulated state with increasing MDSC frequencies (27).

In conclusion, IL-3 is a potent factor for *in vitro* generation of M-MDSC from mouse BM. Their phenotype and function are highly similar to GM-CSF generated M-MDSC. As a major difference, IL-3 generated M-MDSC express higher levels of Arg1 at day 3 of culture as compared with GM-CSF M-MDSC, which can be further up-regulated after stimulation without affecting the iNOS levels. The higher Arg1 expression is also reflected by its elevated functional use as a suppression molecule for T cell proliferation. Our data provide novel insights about IL-3 as an inducer of M-MDSC, which paves the way for further molecular investigations on their signaling, but also about their role *in vivo* in type 2 diseases such as allergies and helminth infections.

## Materials and methods

### Mice

C57BL/6J (Charles River) mice were bred in our animal facilities, kept under specific pathogen–free conditions, and used at an age of 7–12 weeks. All animal experiments were performed according to the German animal protection law as well as after approval and under control of the local authorities.

### *In vitro* generation of IL-3 and GM-CSF MDSC, stimulation, and proliferation measurement of progenitor cells

MDSC were generated from murine BM by culturing fresh cells in the presence of 10 ng/ml of recombinant murine GM-CSF or IL-3 (Peprotech) for 3 days. Alternatively, supernatant from a GM-CSF producing cell line (29) was used at the same activity. Cells were plated at a concentration of 3x10^6^ cells/ml in 10cm petri dishes (Corning) in 10ml R10 medium (500ml RPMI 1640 (PAA Paching Austria), 10% heat-inactivated sterile filtered fetal calf serum (PAA), 100U/ml penincillin (PAA), 100μg/ml streptomycin (PAA), 2mM L-glutamine (PAA), 50mM β-mercaptoethanol (Sigma-Adrich)). At day 3, cells were transferred to a 24-well plate at a concentration of 1 x 10^6^ cells per well in a total volume of 1ml, and were stimulated overnight by either 0.1µg/ml LPS (from *E. coli*, Sigma-Aldrich) + 100U/ml recombinant murine IFN-γ (Immunotools) or 5µg/ml Zymosan A (from *S. cerevisiae*, Sigma-Aldrich) + 100U/ml recombinant murine IL-4 (Peprotech), or were left untreated. At day 4, the cells were harvested and used for flow cytometry analysis. To measure the influence of the growth factors in the proliferation of GMP and MDP progenitors, fresh BM cells were cultured for 3 days in the presence of 10 ng/ml of recombinant murine GM-CSF or IL-3. Proliferation was measured after 3 days by flow cytometry as frequency of the proliferation marker Ki67.

### Generation of BM-derived MC with IL-3

Fresh BM cells were cultured with 10ng/ml IL- 3 at a concentration of 1x10^6^ cells/well in a 6-well plate in a total volume of 6ml. At day 4, 3ml supernatant per well was discarded, and 3ml fresh R10 with 1% IL-3 was added for feeding. The same procedure was repeated at day 7. After ten days, cell suspensions were washed and plated again with IL-3 as described above. At day 15, the cells were harvested, washed, and analyzed by flow cytometry.

### Flow cytometry

The murine directly conjugated antibodies CD11b-PerCP-Cy5.5/-APC/-Alexa Fluor 700 (M1/70), CD49a-PE/-PerCP-Cy5.5 (HMα1), CD11c-Pacific Blue/-PE-Cy7 (N418), Ly6G-Fitc/-PerCP-Cy5.5/-APC-Cy7 (1A8), Ly6C-Alexa Fluor 647/-Alexa Fluor 488/-Brilliant Violet 510 (HK1.4), CXCR4-Pacific Blue (L276F12), CD4-APC/-PerCP-Cy5.5/-Pacific Blue/-Alexa Fluor 488 (GK1.5), CD8-PerCP-Cy5.5/-APC/-Alexa Fluor 488 (53-6.7), Ki67-PE (11F6), Sca-1-Brilliant Violet 510 (D7), c-Kit-PE-Cy7 (2B8), CD16/32-PerCP-Cy5.5 (93), CD34-APC-Cy7 (HM34), Terr119-Alexa Fluor 488 (TER-119), B220-Alexa fluor 488 (RA3-6B2), FcεR Iα-Alexa Fluor 700 (MAR-1), M-CSFR-Biotin (AFS98), Streptavidin-PE were all purchased from Biolegend. iNOS-PE (CXNFT), Arginase I-APC (A1exF5), pmTOR-eFluor 450 (MRRBY), pAkt-APC (SDRNR), pS6-eFluor 710 (cupk43k) and Fixable Viability Dye-eFluor 780 (#65-0865) were obtained from eBioscience. pSTAT1-Aexa Fluor 647 (pY701), pSTAT3-Alexa Fluor (pY705), pSTAT5-Alexa Fluor 647 (pY694) were obtained from BD Biosciences. IL-3Rα-PE (#151231), GM-CSFRα-PE (#698423), CCR2-PE (#475301) were purchased from R&D Systems. IL-4Rα-PE (mIL4R-M1) was obtained from BD PharMingen. Cells were stained in PBS containing 0.1% BSA, 0.1% sodium azide, and 5% fetal bovine serum in the dark for 30 minutes on ice. For intracellular iNOS and Arg1 detection, cells were first labeled for surface markers and then washed and subsequently fixed with 2% PFA for 20 minutes at room temperature. Cells were then permeabilized and stained with Perm buffer 1X (eBioscience) for 1 hour at room temperature together with the marker-specific antibody. Samples were washed once in Perm buffer, resuspended in FACS buffer and measured with a FACS Calibur, LSR II (Becton Dickinson) or Attune NxT (Thermo Fisher Scientific). Results were analyzed with FlowJo Software (Tree Star).

### Cytospins, cell staining and microscopy

For visualization of granulocytic and monocytic cell morphology, IL-3 or GM-CSF cultured BM day 3 cells were collected and washed. To allow their fixation on glass slides, 5-20x10^4^ cells were deposited into cytospin chambers and centrifuged at 600rpm for 10 **min**, after which they were left to dry. Ultimately, the slides were plunged in 3 different Diff-Quick solutions - Diff-Quick Fix, Diff-Quick I and Diff-Quick II. Finally, the slides were washed, air-dried, and pictured with PrimoStar and Labscope softwares (Carl Zeiss, Jena, Germany).

### Enzyme-linked immunosorbent assay

BM cells were cultured with GM-CSF or IL-3 for 3 days as described above, after which they were stimulated with LPS+IFN-γ overnight. On day 4, culture supernatants were collected, and IL-1β, TNF and IL-10 production was quantified by ELISA (Biolegend) according to the manufacturer’s instructions.

### Suppressor assay

Bulk spleen cells were plated in 96-well plates at a concentration of 200.000 cells/well in 200µl R10 and stimulated with soluble aCD3/aCD28 antibodies at a final concentration of 2.5µg/ml. *In vitro* GM-CSF- or IL-3-generated MDSCs were titrated and added to the splenocytes at different ratios. The cells were co-cultured over 3 days and proliferation was measured by flow cytometry *via* Ki67 detection separately in CD4- and CD8-stained T cell subsets. iNOS inhibitor N^G^-Methyl-L-arginine acetate salt (L-NMMA, 500 μM, MilliporeSigma) and Arginase-I inhibitor Nω-hydroxy-nor-arginine (nor-NOHA, 50μg/ml, Cayman Chemical) were added to the co-cultures when required.

### Statistics

Figures were created and statistics were calculated using Excel or Prism 9 (GraphPad Software) programs. Details on the statistical tests used are provided in the figure legends and include the student’s t-test and ANOVA. P values of less than 0.05 were considered significant.

## Data availability statement

The original contributions presented in the study are included in the article/**Supplementary Material**. Further inquiries can be directed to the corresponding author.

## Author contributions

AA, SS and MH performed the experiments and analyzed the data. AA and ML designed the experiments, prepared the figures and wrote the paper. All authors contributed to the article and approved the submitted version.
