# Supplementary material for: M-MDSC in vitro generation from mouse bone marrow with IL-3 reveals high expression and functional activity of arginase 1
Source: Front Immunol. 2023 May 19;14:1130600. doi: 10.3389/fimmu.2023.1130600 (PMC10235456; doi:10.3389/fimmu.2023.1130600)
Supplement: Supplementary file 1 [file DataSheet_1.pdf]

Supplementary Material

M-MDSC *in vitro* generation from mouse bone marrow with IL-3 reveals high expression and functional activity of arginase 1

Arpa Aintablian[1], Sandra Strozniak[1], Marion Heuer[1], Manfred B. Lutz[1*]

[1] Institute for Virology and Immunobiology, University of Würzburg, Würzburg, Germany.

[*] **Correspondence:** Manfred B. Lutz, m.lutz@vim.uni-wuerzburg.de

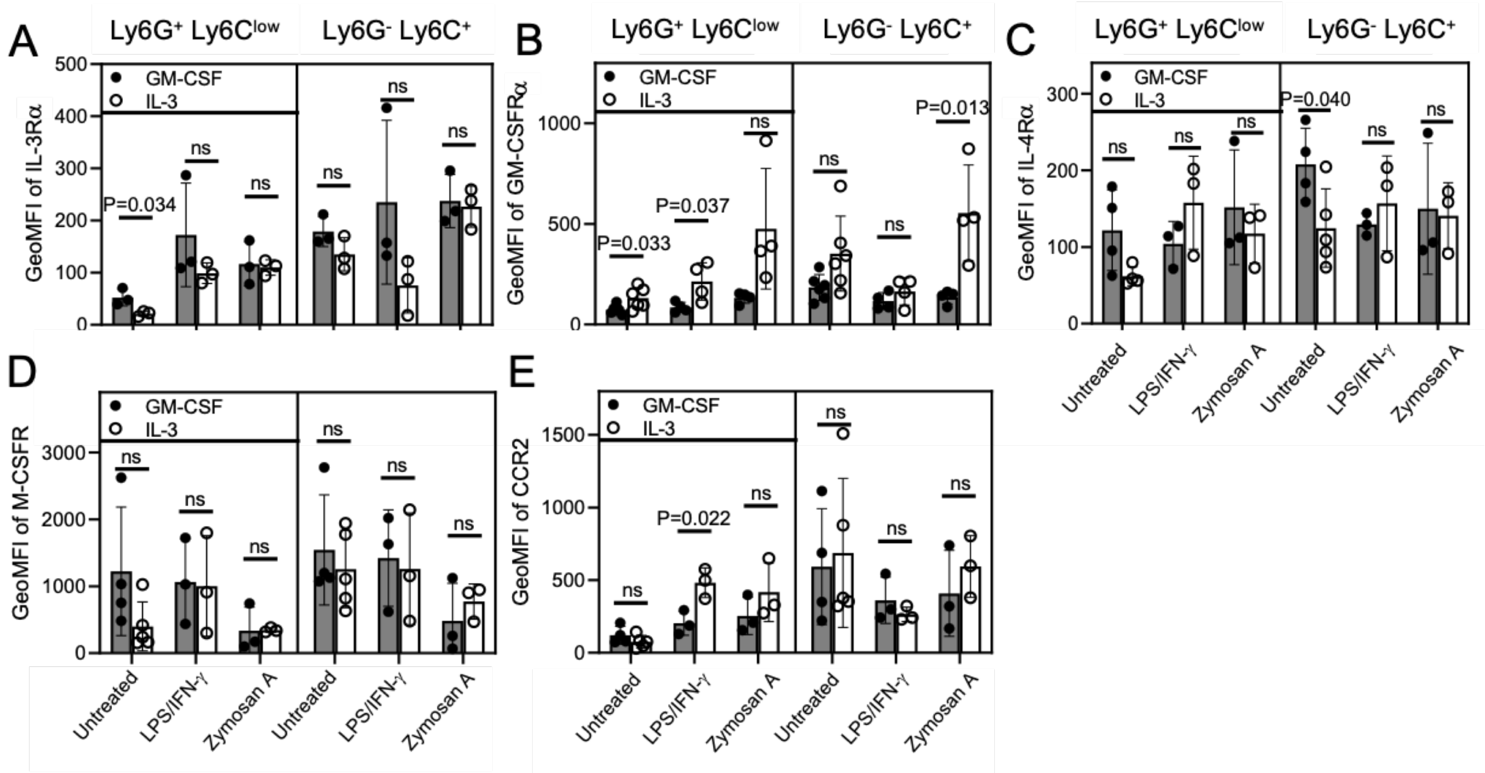

Supplementary Figure 1

Surface expression of cytokine and chemokine receptors is similar between unstimulated and stimulated BM cultures with IL-3 or GM-CSF.

A-E. BM cells cultured in GM-CSF or IL-3 were transferred to a 24-well plate at day 3 and stimulated overnight with either 0.1µg/ml LPS + 0.01ng/ml IFN-γ or 5µg/ml Zymosan A or remained unstimulated. At day 4, flow cytometry was performed to detect the surface expression of the indicated receptors per cell within $CD11b^+$ cells and as further indicated. Statistics compare GM-CSF with IL-3 cultures, by unpaired, two-tailed t-test, n=3-6 independent experiments. Not significant (ns).

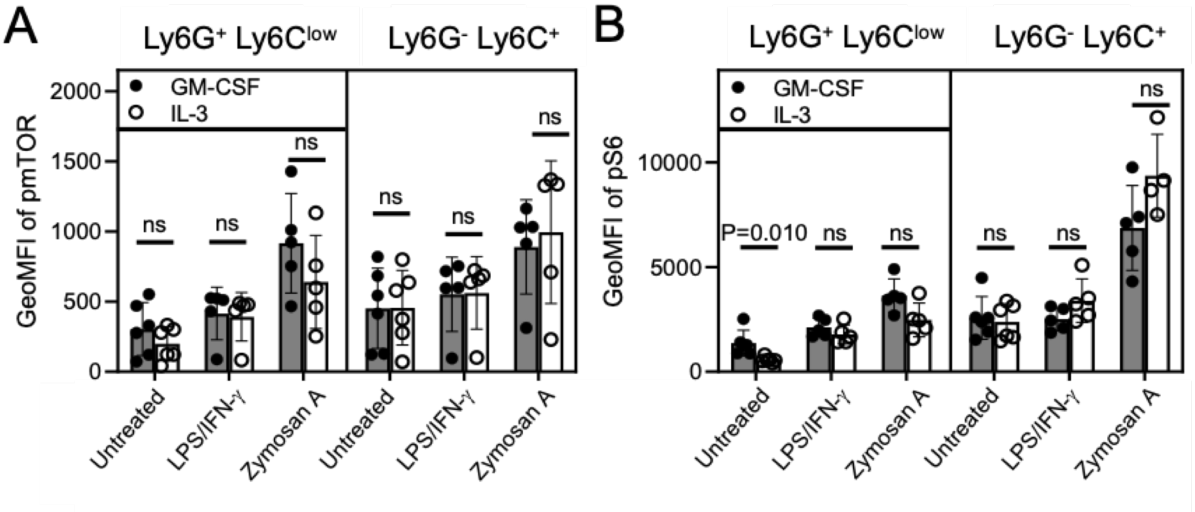

Supplementary Figure 2

Expression of signaling molecules is similar between unstimulated and stimulated BM cultures with IL-3 or GM-CSF.

A,B. BM cells cultured in GM-CSF or IL-3 were transferred to a 24-well plate at day 3 and stimulated overnight with either 0.1µg/ml LPS + 0.01ng/ml IFN-γ or 5µg/ml Zymosan A or remained unstimulated. At day 4, flow cytometry was performed to detect the intracellular expression of the indicated molecules per cell within $CD11b^{+}$ cells and as further indicated. Statistics compare GM-CSF with IL-3 cultures, by unpaired, two-tailed t-test, n=3-6 independent experiments. Not significant (ns).

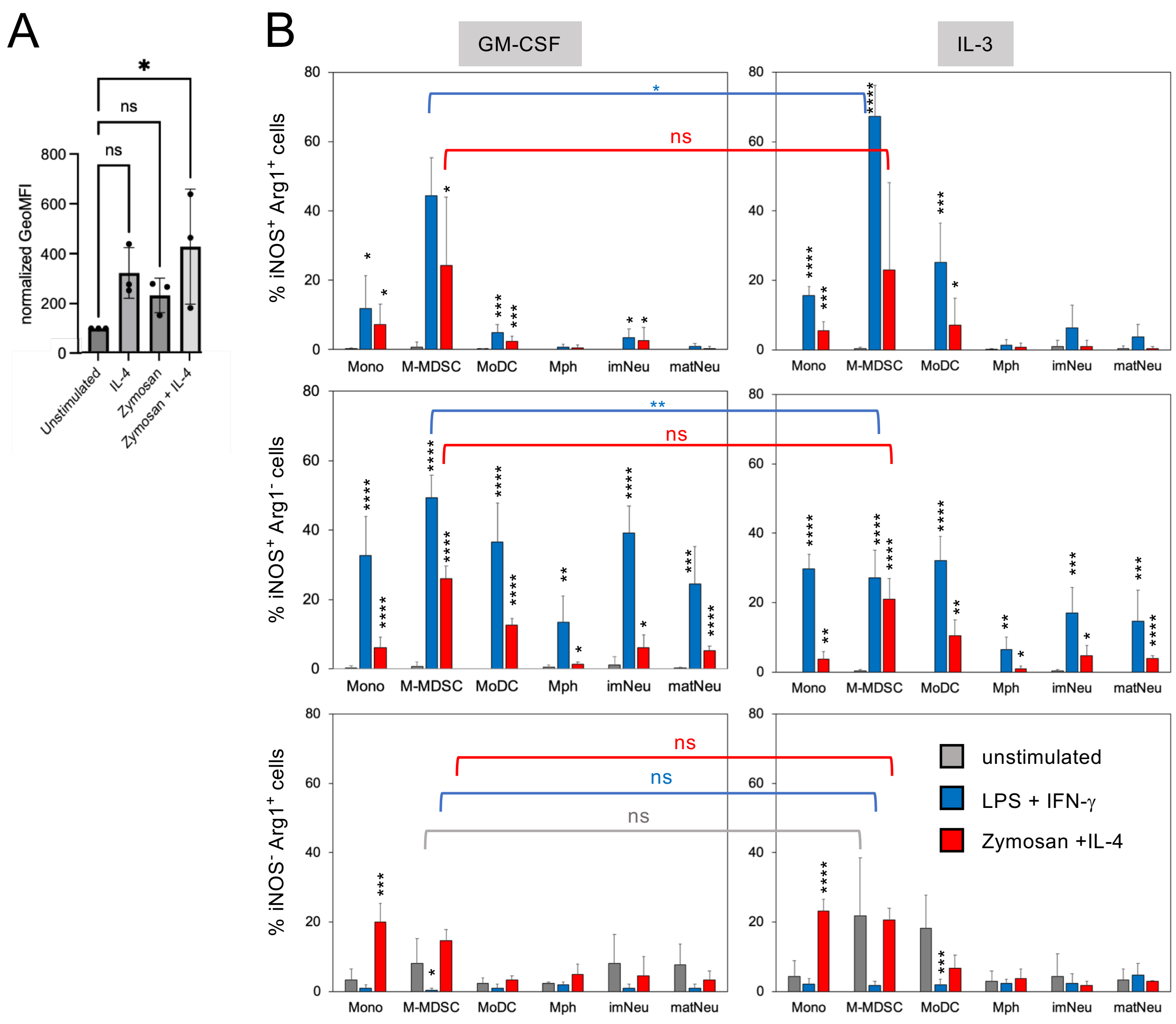

Supplementary Figure 3.

IL3-MDSC cultures develop higher frequencies of iNOS- Arg1+ M-MDSC without activation and generate higher frequencies of iNOS+Arg1+ M-MDSC after LPS/IFN-γ stimulation, while NOS+Arg1- M-MDSC show lower frequencies.

A. BM cells cultured in IL-3 for 3 days were transferred to a 24-well plate at day 3 and stimulated overnight as indicated with 5µg/ml Zymosan and/or 100U/ml IL-4 or remained unstimulated. Gating strategy used for flow cytometry as in Fig. 2A. Arg1 GEO-MFI values were normalized to unstimulated cells and only data for M-MDSC are displayed. Statistics: One-way ANOVA, multiple comparisons; n=3 independent experiments. Not significant (ns), * p<0.05. **B.** BM cells cultured in IL-3 or GM-CSF for 3 days were transferred to a 24-well plate at day 3 and stimulated overnight with either 0.1µg/ml LPS + 0.01ng/ml IFN-γ or 5µg/ml Zymosan + 100U/ml IL-4 or remained unstimulated. Gating strategy used for flow cytometry as in Fig. 2A. Monocytic and granulocytic cell subsets are shown as indicated. Flow cytometry example staining at day 4 showing the intracellular expression of iNOS and Arg1 for all cell types. An extract of this figure is also shown as Fig. 5B. Statistics: unpaired, one-tailed t-test compared to unstimulated cells (black, only significant differences indicated); or unpaired, two-tailed t-test comparing GM-CSF and IL-3 cultures (blue, red), n=4 independent experiments. Not significant (ns), * p<0.05; ** p<0.01; *** p<0.001; **** p<0.0001.

Suppl-Fig. 4

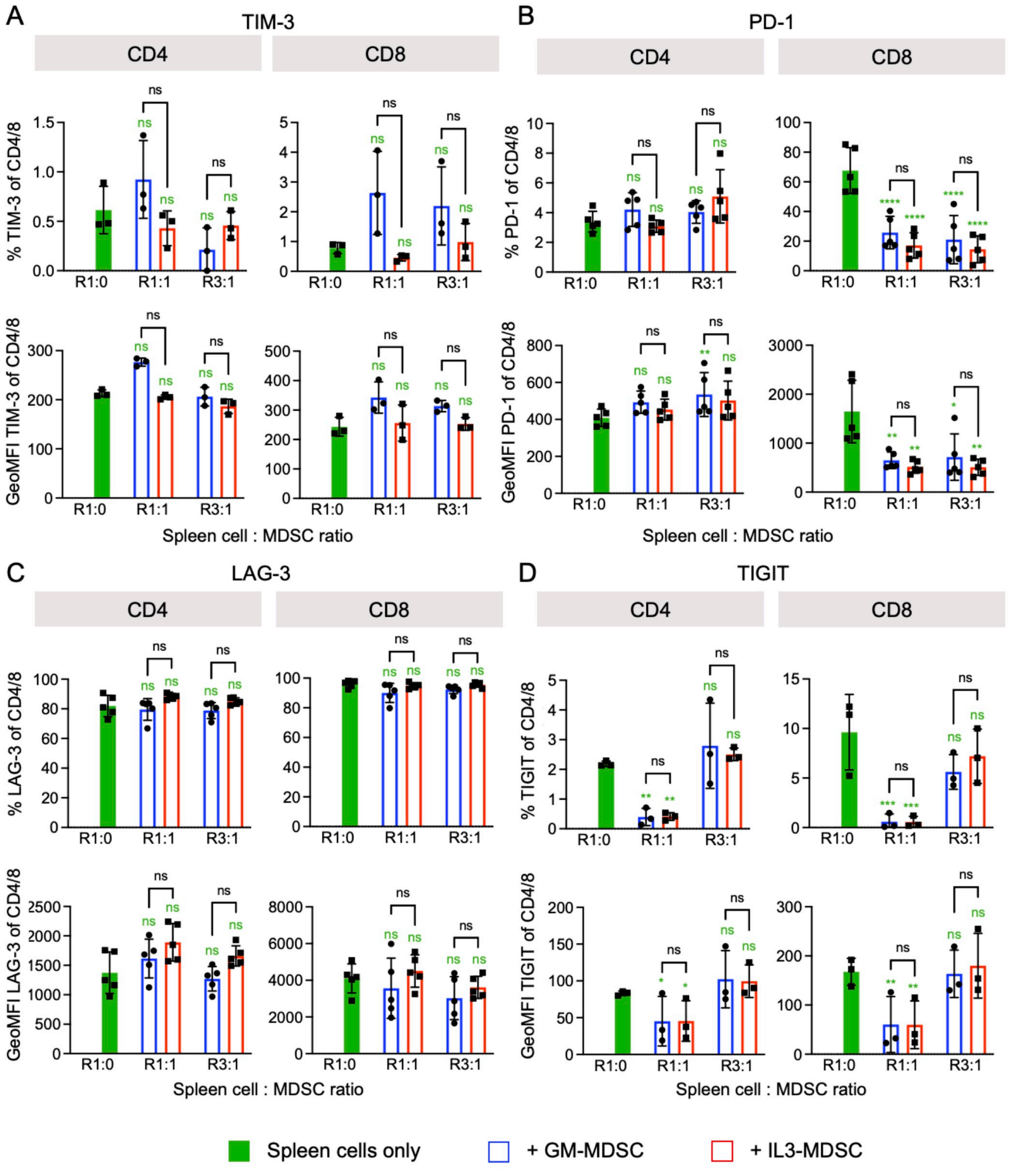

Suppl. Figure 4.

CD4 and CD8 T cells do not upregulate exhaustion markers following suppression by GM- or IL-3 generated M-MDSC.

A-D. Bulk spleen cells were stimulated with anti-CD3/CD28 antibodies and cultured for 3 days in the presence of GM-CSF- or IL-3-generated MDSC at the indicated ratios. T cell exhaustion is measured by flow cytometry and depicted as frequency and GeoMFI of exhaustion markers TIM-3, PD-1, LAG-3 and TIGIT within CD4+ or CD8+ T cell subsets. Statistics was performed with two-way ANOVA and Tukey's post test as indicated by the lines (black, blue, red) between the respective bars, and in green comparing to 'spleen cells only'. n=3-5 independent experiments. Not significant (ns); * p<0.05; ** p<0.01; *** p<0.001; **** p<0.0001.
